# Commercial determinants of mental ill health: An umbrella review

**DOI:** 10.1371/journal.pgph.0003605

**Published:** 2024-08-28

**Authors:** Kate Dun-Campbell, Greg Hartwell, Nason Maani, Alice Tompson, May CI van Schalkwyk, Mark Petticrew

**Affiliations:** 1 Department of Health Services Research and Policy, London School of Hygiene and Tropical Medicine, London, United Kingdom; 2 Department of Public Health, Environments and Society, London School of Hygiene and Tropical Medicine, London, United Kingdom; 3 School of Social and Political Science, University of Edinburgh, Edinburgh, United Kingdom; York University, CANADA

## Abstract

Mental ill health has complex and interrelated underlying causes, with wider determinants of health often overlooked as risk factors. The ‘commercial determinants of health’ are gradually receiving more attention and recognition but there is a relative lack of awareness of the commercial determinants of *mental* health. This aim of this umbrella review was to synthesise systematic review level evidence for the association between commercial determinants and mental health outcomes. This umbrella review included evidence from high, middle, and low-income countries. We included terms related to broader commercial activities and terms focused on six key unhealthy commodities (tobacco, alcohol, ultra-processed foods, gambling, social media, fossil fuels) and the impacts of fossil fuel consumption (climate change, air pollution, wider pollution). We included 65 reviews and found evidence from high quality reviews for associations between alcohol, tobacco, gambling, social media, ultra-processed foods and air pollution and depression; alcohol, tobacco, gambling, social media, climate change and air pollution with suicide; climate change and air pollution with anxiety; and social media with self-harm. There was a lack of evidence examining wider practices of commercial industries. Our umbrella review demonstrates that by broadening the focus on commercial determinants, the influence of commercial products and activities on mental ill health can be better understood. The lack of research examining broader commercial practices on mental ill health is an area that should be addressed. Our review highlights the existing base of high-quality evidence for many of these unhealthy commodities’ impacts on mental ill health and indicates that commercial determinants is a valuable framework for understanding the drivers of mental ill health.

## Introduction

Factors that determine mental health are both complex and interrelated [1]. Globally, around 1 in 8 people are living with a diagnosed mental health disorder [1] - although this is likely an underestimate of the true proportion of people living with mental ill health. Existing research shows a focus on individual susceptibility and life experiences such as childhood trauma. This can overlook the context in which these experiences occur including the ways in which wider social, political, economic and commercial forces shape mental health and inequalities [2, 3]. Recent research, reflecting a growing interest in the social determinants of health (SDOH), has focused on these wider factors – such as household income, employment and housing [4, 5]. The commercial determinants of mental health (CDMH) in particular, have not yet been afforded a similar level of attention [6].

The commercial determinants of health (CDOH) can be thought of as “the systems, practices, and pathways through which commercial actors drive health and equity” [7]. This includes both the direct and indirect effects of the consumption of produced commodities - such as tobacco, alcohol, fossil fuels and unhealthy foods - and the drivers of consumption such as marketing and advertising [8]. In addition to analysis of specific unhealthy commodities, CDOH research also includes analysis of the role of commercial actors in shaping the political, structural, and cultural environments which affect health [9].

These commercial influences can impact not just physical but also mental health, since unhealthy commodity products directly affect and/or harm mental health [10–12]. There is already an evidence base, for instance, for the impact of alcohol consumption on depression and suicide [13] and smoking on depression and anxiety [14]. Yet the effects are also indirect; for example, the producers of harmful commodities frequently adopt framings of individual responsibility to place the blame for product harms on individuals themselves. This is often done though ‘responsibility’ campaigns and slogans such as “Gamble/Drink Responsibly” [15].

Despite the evidence for impacts of unhealthy commodity consumption on health outcomes, existing frameworks for the social determinants of health generally do not consider commercial determinants; nor do they typically include mental health [16]. There is a strong case for drawing together the existing evidence on mental ill health and commercial determinants. This is of value both for informing the further development of existing SDOH frameworks and identifying points at which to intervene on the CDMH.

This umbrella review therefore aimed to synthesise systematic review evidence on the effects of commercial determinants on mental ill health outcomes to map and identify gaps in the existing evidence base.

## Methods

The review was developed according to the Preferred Reporting Items for Systematic Review and Meta-Analyses (PRISMA) guidelines and the protocol registered on PROSPERO 2022 CRD42022320288 [17].

### Eligibility criteria

Inclusion criteria were:

- Population: High, middle, and low-income countries.
- Intervention/exposure: Commercial determinants of mental health, including market strategies and non-market strategies, across six key unhealthy commodities (tobacco, alcohol, ultra-processed foods, gambling, social media, fossil fuels), and the results of fossil fuel consumption (climate change, air pollution, wider pollution).
- Outcome: Mental health outcomes, anxiety, depression, self-harm, and suicide. Any type of measure was included (e.g., self-reported or assessed by a clinician).
- Study design: Full-text articles, in the English language, between 2012-2023 (limited to the past 10 years to ensure a manageable number of results; and in the case of social media to ensure there were sufficient studies), systematic review, meta-analysis, narrative review, scoping review.

### Exclusion criteria

Studies that examined mental well-being, or severe mental illness, including bipolar disorder, schizophrenia, and other psychotic disorders, and eating disorders were excluded. As noted- for reasons of feasibility- the review focused on six industries with major relevance to health: tobacco, alcohol, social media, ultra-processed foods, gambling, and fossil fuel products. We also chose to only include adverse impacts from large-multinational manufacturers [18]. Although the private sector often undertakes important social functions aligned with health benefits, these positive health impacts are already incentivised through the commercial incentives of profit-seeking, just as negative health impacts are. As Maani et al. have therefore argued [15], focusing research on areas where profit and health are misaligned is likely to contribute to greater short-term net health benefits. Similarly, the most significant scientific insights can be expected by focusing on the largest commercial entities rather than the manifold small and medium organisations than constitute a far smaller fraction of overall commercial impacts on health.

### Search strategy

**Fig 1:**
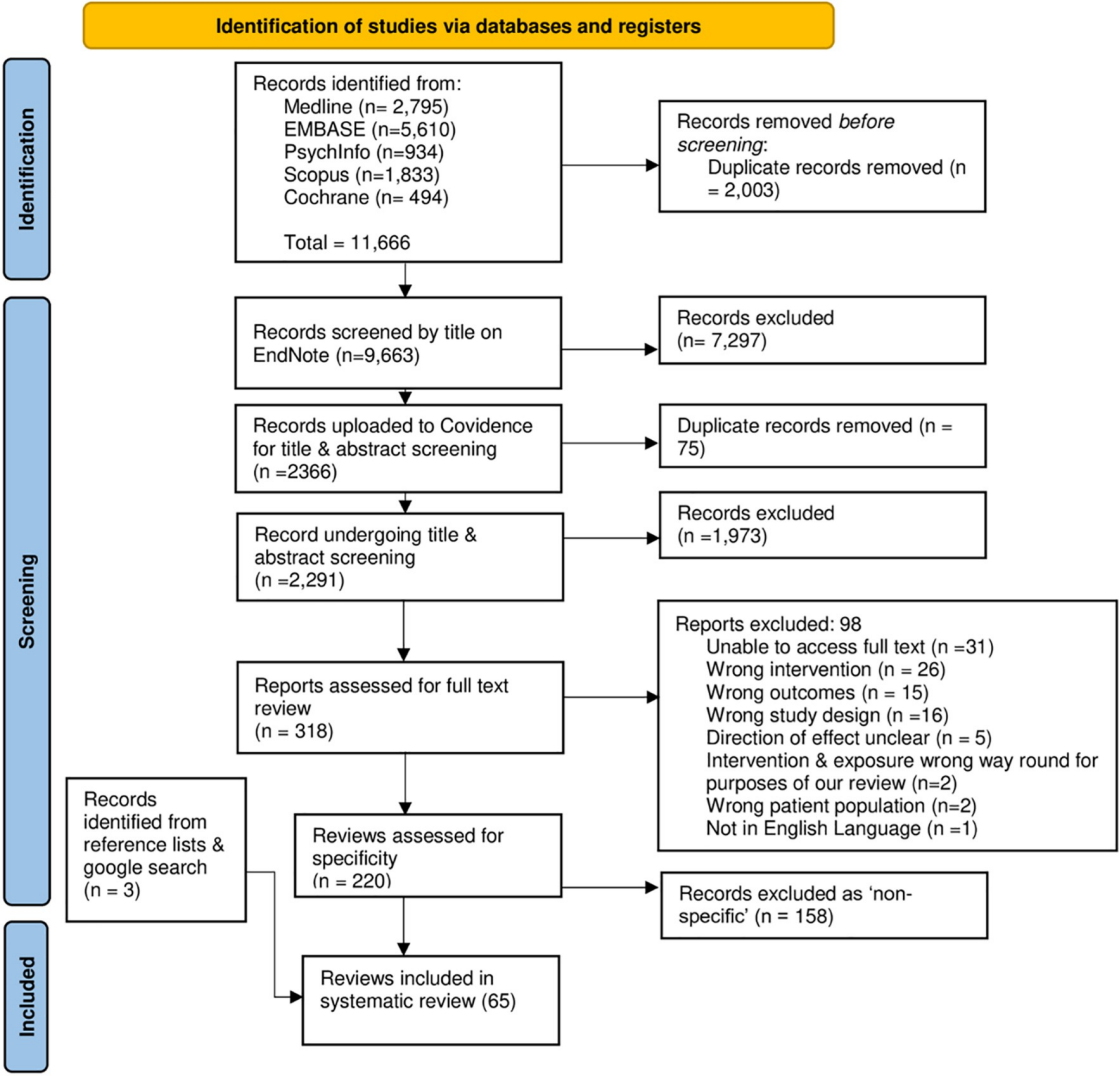
PRISMA flow diagram [21]. PRISMA outlining the number of reviews found for each database searched, the number excluded, and the final number included.

The literature search was developed in Medline and adapted for use in other databases (see S1–S3 Figs for full search strategy). Search terms relating to the influence of commercial actors were developed based on Lee et al.’s (2022) ‘Conceptual Framework for the Study of the Commercial Determinants of Health’ – including market and non-market strategies [8]. KDC ran pilot searches to develop additional terms, then conducted searches of Medline, PubMed, PsychInfo, Scopus and the Cochrane database on 28^th^ March 2022 (Repeat searches were run on 7^th^ August 2023). See Fig 1 for PRISMA.

### Study selection

One reviewer (KDC) conducted initial screening by title. Two reviewers (KDC and GH) screened reviews in Covidence [19] by title and abstract; each screened 50% of the reviews with a sample of 100 screened by both to check agreement. This sample had high inter-rater reliability with Cohen’s Kappa of 0.8 (strong agreement). The full-text screening was undertaken by two independent reviewers (KDC and GH) using Covidence. We discussed disagreements between the reviewers, with high reliability (Cohen’s Kappa = 0.71, indicating substantial agreement). At this stage of the search, we had an unmanageable volume of reviews so decided to limit the included reviews to those that focused specifically on our exposures and outcomes of interest (as opposed to prevalence studies examining a very wide range of exposures or outcomes, of which only one or a small part would be relevant).

### Data collection process and data items

We extracted information on the name of the review, authors, date, number of studies included, type of studies included (e.g., cohort, cross-sectional), measure of exposure, measure of outcome, pooled effect estimate (if available), summary of results, proposed mechanism for effecting mental health and funding. One reviewer (KDC) extracted the data, and results were grouped for each industry and sent to a second reviewer (GH) for review.

### Quality assessment

We used the Scottish Intercollegiate Guidelines Network (SIGN) checklist for systematic reviews and meta-analyses [20] to assess each study’s quality.

Quality was rated as ‘low’, ‘acceptable’ and ‘high’ using the criteria set out in the guidance notes - “High quality (++): Majority of criteria met. Little or no risk of bias. Acceptable (+): Most criteria met. Some flaws in the study with an associated risk of bias. Low quality (-): Either most criteria not met, or significant flaws relating to key aspects of study design. Reject (0): Poor quality study with significant flaws. Wrong study type. Not relevant to guideline” [20]. For example, a review was unable to achieve a ‘high’ rating if the review did not assess the quality of its included studies. We included all reviews deemed to be of “high” and “acceptable” quality. Low-quality reviews were only included if they contained an exposure or outcome which was not well represented in the sample.

### Synthesis of results

We used narrative synthesis to combine the findings across the included reviews. It was decided during our study design that meta-analysis was not appropriate due to heterogeneity in the measurement of exposures and outcomes across included studies – this is outlined in our protocol on PROSPERO 2022 CRD42022320288 [17].

## Results

Our search returned 11,666 reviews across the five databases. Once duplicates were removed, 9,663 records were screened by title, of which 2,366 were uploaded to Covidence [19] for abstract screening. We then assessed 318 full texts for eligibility. This left a total of 220 reviews, of which 158 were excluded for being non-specific (as per ‘study selection’ above).

**Fig 2:**
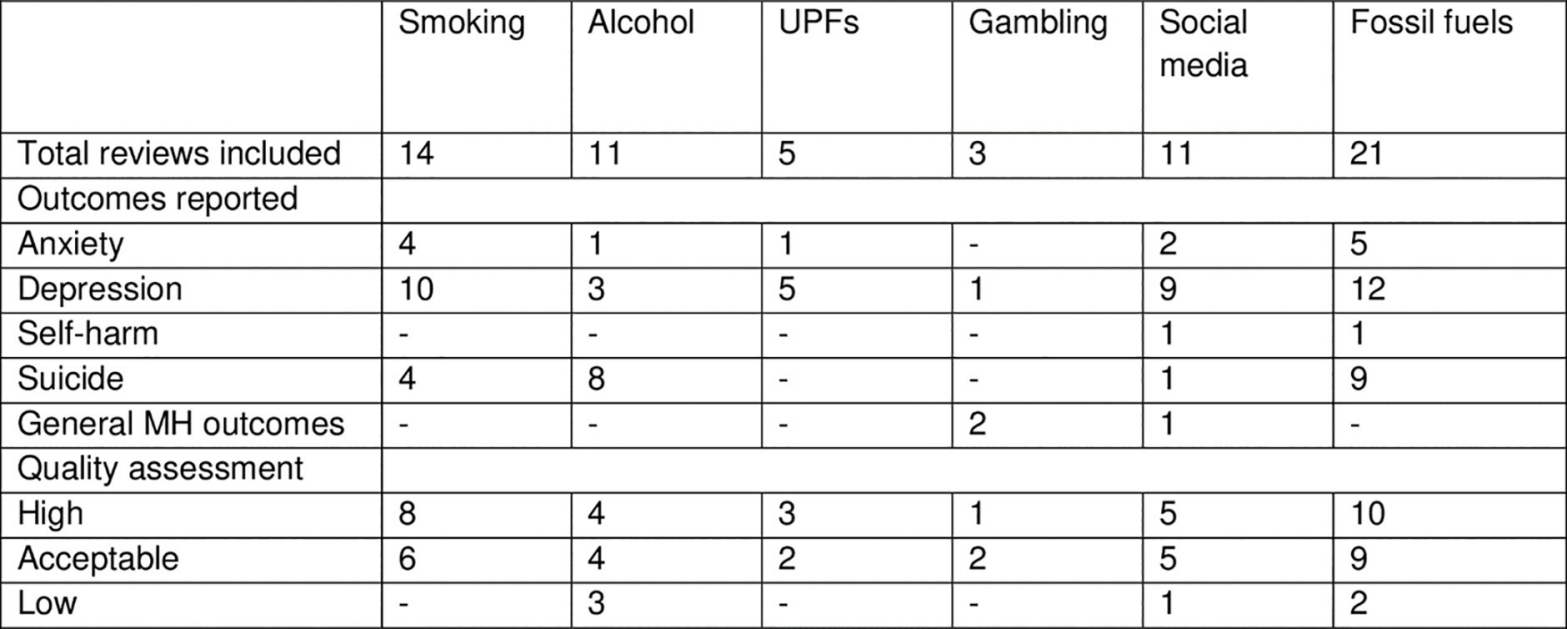
Summary of review characteristics. A summary table of the number of reviews included by each exposure and outcome and the number of reviews of each quality. Note numbers may not total the number of reviews included as some reviews reported multiple outcomes.

**Fig 3:**
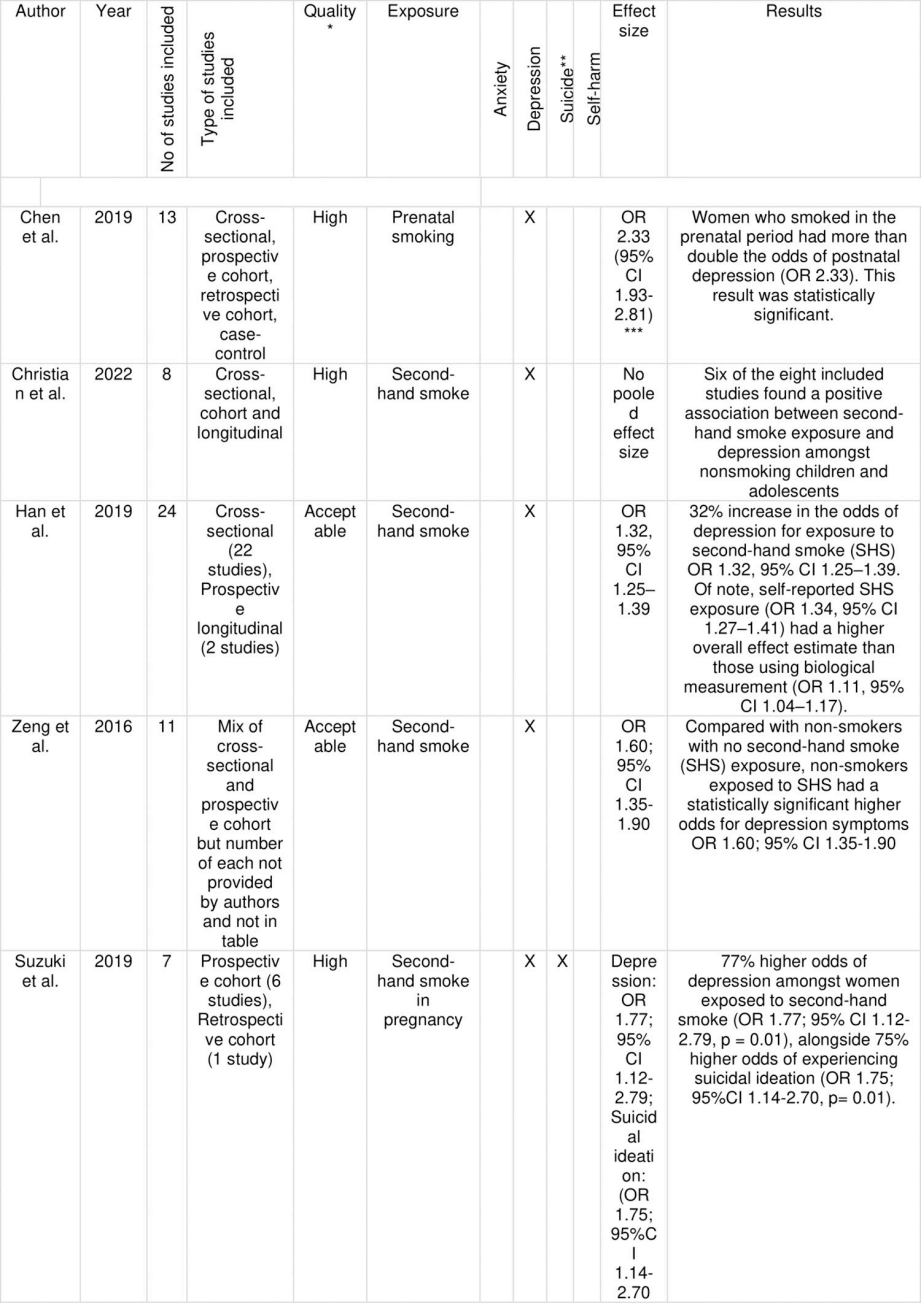
Review characteristics and summary of findings. Outlining the characteristics and main findings of each of the reviews included in our umbrella review. * As rated by SIGN criteria ** Includes suicidal ideation, planning, attempt and completed suicide *** Rounded to 2 decimal places by KDC **** Note this is the pooled effect using the fixed effects model as reported by the authors - given the high heterogeneity, it could be more appropriate to use the random effects model CI: Confidence interval; OR: Odds Ratio; PM: Particulate Matter; RR: Risk Ratio; HR: Hazard Ratio.

We included 65 reviews in the final synthesis, see Fig 2 for summary of reviews and Fig 3 for review characteristics and main findings. Fourteen reviews examined the impact of smoking on mental health outcomes. Eight were rated as high and six as acceptable quality. We included eleven reviews of alcohol consumption and mental health outcomes. We rated four as high, four as acceptable, and three as low-quality. Five reviews examined ultra-processed food as an exposure; we assessed three as high and two as acceptable quality. Three reviews examined the impact of gambling on mental health outcomes, we assessed one as high and two as acceptable quality. We identified 11 reviews that included social media as an exposure. Of these, we rated five as high, five as acceptable, and one as low-quality. We identified 21 reviews related to fossil fuel products and their impacts. 10 focused on air pollution, eight on climate ‘change’, and four on pesticides. We rated 11 reviews as high, nine as acceptable and one as low-quality.

### Smoking and mental ill health

We included fourteen reviews examining the impact of smoking on mental health outcomes [22–35].

There was evidence from high quality reviews for associations between second-hand smoke with depression in children and adolescents, second-hand smoke exposure in pregnancy and smoking in pregnancy with postnatal depression, smoking in pregnancy with suicidal ideation, and smoking and suicide. There was evidence from high quality reviews that smoking cessation improved symptoms of depression, anxiety and mixed anxiety and depressive disorder.

Of the included studies examining anxiety, there was evidence from an acceptable quality review that daily smokers had 5 times the odds of panic disorder at age 24 than non-daily smokers (Odds Ratio (OR) 5.1; 95% Confidence Interval (CI) 2.4-10.5) [31]. The same review reported 15 times the odds for smokers smoking more than 1 pack per day, than under 1 pack, for generalised anxiety disorder (OR 15.58; 95%CI 2.31-105.14). Another acceptable quality review reported two out of four included prospective observational studies found smoking status was predictive of anxiety [28].

One high [23] and three acceptable quality [24, 25, 30] reviews examined the association between smoking and depression. In addition, one review of prospective studies found a statistically significant 62% higher odds of depression at follow up among smokers vs never smokers. (OR 1.62 95%CI 1.10-2.40). A smaller effect size was found(32%), when only prospective studies with both baseline and follow up data were included (OR 1.32, 95%CI 1.02-1.71) [30].

### Exposure to secondhand smoke

Three reviews examined second-hand smoke (SHS) [23–25]. One focused on children and adolescents specifically [23]; in this review there was no pooled effect size but six of the included eight studies found a positive association and a dose-response relationship between SHS exposure and depressive symptoms in children and adolescents (SHS exposure in the home and in public places were included as measures). Two included acceptable quality reviews found statistically significant associations between exposure to SHS and depression - one found 32% higher odds of depressive symptoms in those exposed to SHS (OR 1.32, 95%CI 1.25–1.39) and evidence of a dose-response effect [24], the other 60% higher odds of depressive symptoms with exposure to SHS (OR 1.60, 95%CI 1.35–1.90) [25].

### Smoking in pregnancy

Two high-quality reviews examined smoking in pregnancy and reported statistically significant results. One found that engaging in prenatal smoking was associated with more than twice the odds of postpartum depression [22] and the second found that women exposed to second-hand smoke during pregnancy had 77% higher odds of postpartum depression and 75% higher odds of antenatal suicidal ideation [26]. In addition, one review [28] found 37 of the 51 studies they included (73%) reported that smoking increased the risk of subsequent depression. Among included prospective observational studies two out of four found smoking status was predictive of anxiety.

Three high-quality systematic reviews reported an association between smoking and suicide outcomes (including suicidal ideation, planning, attempt, and death by suicide) [27, 29, 32]. One review of prospective cohort studies found an 81% higher risk for completed suicide in those who were current smokers at the time of death vs non-smokers (Relative risk (RR) 1.81; 95%CI 1.50-2.19), with the risk of suicide increasing by 24% for each additional ten cigarettes smoked daily. Both these findings were statistically significant [29]. A second high-quality review, also including only prospective cohort studies, found that, compared with never-smokers, current smokers experienced 2.4 times the risk of death by suicide (RR 2.41; 95%CI 2.08-2.80) and nearly twice the risk of suicidal ideation (RR 1.84; 95%CI 1.21-2.78), with both findings statistically significant [27]. The third review [32] replicated these findings for suicidal ideation and death by suicide, with statistically significantly higher odds for current vs non-smokers - current smokers experienced more than twice the odds of suicidal ideation (OR 2.05 95%CI 1.53-2.58), planning (OR 2.36, 95%CI 1.69-30.02) and attempt (OR 2.84; 95%CI 1.49-4.19) and nearly twice the odds of death from suicide (RR 1.83; 95% CI 1.64-2.02).

Three reviews examined the effect of smoking *cessation* on mental health outcomes [33–35]. Two high-quality reviews [34, 35] reported statistically significant reductions in risk of anxiety, mixed anxiety and depression, and depression from baseline smoking to follow-up after cessation. For example, Taylor et al. (2021) [35] found that the strength of the evidence was greatest for a reduction in risk of in mixed anxiety and depression. One acceptable quality review found lower odds of depression amongst those who had stopped smoking vs current smokers (OR 0.63; 95%CI 0.54-0.75) [33].

### Alcohol and mental ill health

We included eleven reviews examining the impact of alcohol on mental health outcomes [36–46]. There was evidence from high quality reviews for associations between alcohol consumption with depression and suicide.

Amongst reviews examining depression, a high-quality systematic review and meta-analysis including only prospective cohort studies [46] found a statistically significant 57% higher risk of subsequent symptoms of depression in people with alcohol use disorder (RR 1.57; 95%CI 1.41-1.76). Examining dose effects, compared with non-heavy drinkers, heavy drinkers had a 13% higher risk of developing later depressive symptoms (RR 1.13; 95% CI 1.05-1.22). One acceptable quality review [39] found that prenatal alcohol exposure (via maternal drinking) was associated with increased depression and anxiety in children aged three years and over in 69% (9/14) of the included studies. One low-quality review examining older adults (≥50 years) [41] found statistically significant increased hazard ratios for depressive symptoms amongst long-term abstainers (Hazard ratio (HR) 1.14; 95%CI 1.08-1.21) and occasional (HR 1.16; 95%CI 1.10-1.21) and heavy alcohol drinkers (HR 1.22; 95%CI 1.13-1.30) when compared with moderate drinkers.

Two high-quality reviews [40, 45] reported statistically significant associations between alcohol consumption and completed suicide. In one review that only included cohort studies, alcohol use was associated with a statistically significantly 74% increased odds (OR 1.74; 95%CI 1.31-2.31) of completed suicide [40]. A second review including cohort, case-control and cross-sectional studies found higher odds of suicidal ideation (OR 1.86; 95%CI 1.38-2.35), three times higher odds of suicide attempt (OR 3.13; 95% CI 2.45-3.81) and higher odds of completed suicide (OR 2.59; 95% CI 1.95-3.23) for people with alcohol use disorder, all statistically significant [45]. Amongst the acceptable quality reviews [36, 37] examining suicide as an outcome, both found statistically significant associations between acute alcohol ingestion and risk of suicide attempt [36], as well as between any alcohol use and suicidal behaviours (ideation, attempt and completed suicide) [37].

Two included reviews looked at population-level impacts on suicidal outcomes. One high quality [42] and one acceptable quality [44] review found that alcohol policies restricting access to alcohol were associated with lower rates of suicide at the population level. The high-quality review [42] included “enforcing minimum legal drinking age (MLDA), dram shop laws, restrictions on hours of trading, privatization, outlets, and complete alcohol bans”. It did not include a pooled effect size but reported on studies that showed decreases of 3 suicides per 100,000 and 55.5 per 100,000, and a RR of 0.91 (95%CI 0.76-1.08) [42]. The acceptable quality review [44] included analyses of alcohol price and taxation, minimum legal drinking age laws, outlet density, ‘other alcohol policies’ and evaluations of changes in alcohol policy mix in countries other than the US; again it did not have a pooled effect size but reported lower suicide rates following these policies.

Of the two low-quality reviews with suicide as the outcome, one [43] estimated a greater suicide risk amongst ‘alcohol abusers’ vs the general population. The other [38] found that in 27% of suicide post-mortem samples, the blood alcohol level was above zero.

We did not identify any reviews reporting alcohol’s impact on anxiety in adults.

### Ultra-processed foods and mental ill health

We included five reviews examining the impact of ultra-processed foods (UPFs) on mental health outcomes. There was evidence from high quality reviews for associations between UPF consumption and depression [47–51].

One high quality review [47] conducted a meta-analysis of prospective studies and found a 22% higher risk of subsequent depression associated with ultra-processed food consumption (HR 1.22; 95% CI 1.16-1.28). When including all studies, there was a higher odds of depression and anxiety together (OR 1.53; 95% CI 1.43-1.63) and separately (depression OR 1.44; 95% CI 1.14-1.82, anxiety OR 1.48; 95% CI 1.37-1.59).

One high-quality review [51] found a statistically significant 31% higher risk of depression amongst high consumers of sugar-sweetened beverages when compared with low consumers and with non-consumers (RR 1.31; 95%CI 1.24-1.39). This review also found a dose-response relationship, with an increased risk of 5% for 2 cups per day and 25% for 3 cans per day compared with non-drinkers of sugar sweetened beverages. The second high-quality review [49] found a statistically significant 8% higher risk of depression amongst people who ate red and processed meats vs those who did not (OR 1.08; 95% CI 1.04-1.12).

An acceptable quality review [48] analysed several different diets that posed a potential risk for depressive symptoms. The authors referred to these as “pro-inflammatory” diets and they included “sweets; refined flour; high-fat products; red and processed meat” [48]. The second acceptable quality review [50] examined dietary sugars but did not exclusively focus on sugar-sweetened beverages. These reviews reported no pooled effect sizes and found mixed results, though several included prospective cohort studies reported positive associations between added dietary sugars and subsequent risk of depression.

No identified reviews in this group examined self-harm or suicide as outcomes or specifically focused on anxiety. Several different mechanisms for these associations were proposed by the included reviews including systemic inflammation [48, 50], disruption of the gut microbiota, disrupted dopamine function, insulin resistance, oxidative stress or generation of toxic advanced glycation end-products [51].

### Gambling and mental ill health

We included three reviews examining the impact of gambling on mental health outcomes [52–54]. There was evidence from high quality reviews for associations between gambling with depression, and general mental health outcomes.

One high-quality review [52], which did not calculate pooled effect sizes, reported on a study that found onset of ‘problem gambling’ was significantly associated with nearly double the odds of incident major depressive disorder (Adjusted odds ratio (AOR) 1.98; 95%CI 1.14-3.44), and almost 4 times the odds of any mental disorder (AOR 3.84; 95%CI 1.89-7.79) at 3.5 years of follow-up [52]. This review also included several studies which found no association between gambling and later depression or anxiety. The review reported a 15 times higher standardised mortality ratio (SMR) for death by suicide in 20–74-year-olds who had a gambling disorder, compared with the public. For people aged 20-49, the SMR was even higher at 19.3 [52].

Amongst the acceptable quality reviews, one was not exclusively focused on gambling and mental health and included gaming and conduct problems (which are defined as aggressive or antisocial behaviour that impacts on functioning) in its analysis [53]. Focusing on gambling and depression, this review did not report any pooled effects, but 10 out of the 12 included cross-sectional studies found statistically significant positive associations between problem gambling and depressive symptoms [53]. The second acceptable quality review [54] examined online gambling, including only cross-sectional studies, and found several studies reporting a positive association between online gambling and depressive symptoms [54].

### Social media and mental ill health

We included eleven reviews examining the impact of social media on mental health outcomes [55–65]. There was evidence from high quality reviews for associations between social media with depression, suicidal ideation, and self-harm.

There was mixed evidence for an association between social media use and depression. Of the four high-quality reviews [59–63], one [59] which focused on adolescents (11-18 years) and included only cross-sectional studies found a weak positive correlation (*r* = 0.11 p<0.01). Another high-quality review [60], including mostly cross-sectional studies, reported mixed results - with a general association between social media and depression, but no pooled effect size - and noted potential confounders and methodological issues within the included studies [60]. One high-quality review [61] focusing on young people (10-24 years) examined effect sizes for different measures of online media use (including social media) – with greater effect sizes seen when only including studies that used a measure of ‘addiction’ rather than just time spent. A sub analysis based on media type found the effect size of social media to be significantly smaller than internet use.

Another high-quality review focused on adolescents [61] found a positive association between time spent on social media and depression symptoms – OR 1.60 (95%CI 1.45-1.75) with a stronger association for girls (OR 1.72; 95% CI 1.41-2.09) than boys (OR 1.20; 95% CI 1.05-1.37).

The finding that time spent on social media had a small significant positive association with depressive symptoms was replicated across four reviews – two high and two acceptable quality, with three reporting the same effect size [56, 59, 65]; *r* = 0.11 p<0.01, with time spent on social media accounting for around 11% of the depressive symptoms. Among the acceptable-quality reviews, two found statistically significant positive correlations between social media use and depression (*r* = 0.11, 95%CI 0.086 – 0.13, and *r* = 0.11, 95% CI 0.08 - 0.14, p< 0.001) [56, 65].

The remainder of the acceptable quality reviews including depression found a mix of positive and negative associations between social media use and depression [55, 57, 58] but for most of the reviews, these associations were positive.

There was evidence of an association between social media use and anxiety. An acceptable quality review [58] of studies conducted in China reported a significant positive correlation between the two across four included studies, with bivariate correlations ranging between 0.19-0.56.

A high-quality review [62] found nearly 3 times the odds of suicidal ideation amongst adolescents with “problematic” social media use (a definition was not given for this term), which was statistically significant (OR 2.81; 95%CI 1.72- 4.59) [55]. In addition, non-significant associations were found between high frequency of social media use and suicidal ideation (OR 1.45; 95%CI 0.95-2.23), suicidal plans (OR 1.47; 95%CI 0.33-6.43) and self-harm (OR 2.03; 95% CI 0.79-5.21).

A range of mechanisms for the associations between social media use and mental ill health were described in these studies, with many mentioning the mediating impacts of insomnia and other sleep disorders [60, 62], cyber bullying [62, 64] and sexting [57, 62].

### Fossil fuel products, impact of their use and mental ill health

We included 21 reviews examining the impact of air pollution, ambient temperature increases, and pesticides on mental health outcomes [66–85]. There was evidence from high quality reviews for associations between air pollution with depression, anxiety and suicide; ambient temperature increases with risk of suicide; and pesticides with depression and suicide.

#### Air pollution and mental ill health

We found six high quality reviews examining air pollution and depression [67, 70, 72–74, 81]. The reviews found statistically significant but small associations between short term exposure to particulate matter (PM)10 [65, 72], PM2.5 [67], NO2 [67, 70] SO2 [67], or CO [67] and depression. Two reviews also found associations between long-term exposure to PM2.5 or NO2 and depression [67, 72]. One of the reviews estimated a 10% increased risk in depression per 10μg/m3 increase in long term PM2.5 exposure [73]. While the effect sizes were small, the authors highlighted that the population level exposure contributes to a large burden of mental ill health.

One review focused specifically on the impact of air pollution on perinatal mental health and found an association between PM2.5 and NO2 on postnatal depression [74].

Two acceptable quality reviews [68, 82] and one low-quality one [71] supported these findings. There was a strong association between air pollution and hospital admissions for depression in an acceptable quality review (no effect size calculated) [66].

There was also a general association between air pollution and anxiety in these reviews [73, 76]. A statistically significant positive association was found between long-term air pollution and anxiety in two studies included within one high-quality review (the review did not perform meta-analyses due to the low number of studies) [73].

Three high-quality reviews found positive associations between air pollution and suicide. One of these found small positive associations per increased Inter Quartile Range (IQR) in PM2.5, PM10 and NO2 [75]. The second high quality review found statistically significant pooled effect sizes at days 0-2 per 10μg/m3 increase in PM10 [73]. The third found small positive associations between suicide and each 10μg/m3 increase in mean NO2 at a lag of 1-3 days in mean SO2 at 1-4 days and mean PM2.5 at 1 day [69]. Several mechanisms for this were proposed including reduced respiratory function leading to oxidative stress and hypoxia, the latter of which can in turn lead to depleted levels of serotonin [69]. Other suggested mechanisms were via reduced neurophysiological function, stress response pathways, neuroinflammation, decreased cerebral blood flow, cerebral oedema, and swollen nerve cells [75].

#### Temperature increases and mental ill health

Evidence from high, acceptable, and low-quality reviews found ambient temperature increases to be associated with poor mental health outcomes, including risk of suicide. One high quality review found a 9% increased risk of suicide per increase of 7.1C in temperature [75]. Reviews also showed ambient temperature increases to be associated with mental ill health in adults. One high quality review found that each 1C increase in temperature led to an increased risk of mental health related mortality and morbidity [86].

#### Pesticides and mental ill health

One high quality review looked at the impacts of pesticide exposure on anxiety, depression, and suicide in farmers [86] and found an association between exposure to pesticides and depression and suicide. Two acceptable-quality reviews examined pesticide exposure finding evidence of a positive association between pesticide exposure and depression and suicide [84, 85]. Both reviews noted inconsistencies in the methodological approaches of included studies and mixed findings.

### Wider practices/commercial actions and mental health

We identified two reviews examining impacts of introducing policies focused on reducing consumption in any of these six CDoH areas; these both looked at alcohol policy with suicide as the outcome, as discussed in the alcohol results section. Included policies were changes to alcohol pricing, changes to alcohol availability, changes to drink driving countermeasures, increased taxation, regulation of advertising and anti-alcohol advertising [42, 43]. No other identified reviews examined the wider impacts of commercial practices and actions on mental health.

### Synthesis of results

**Fig 4:**
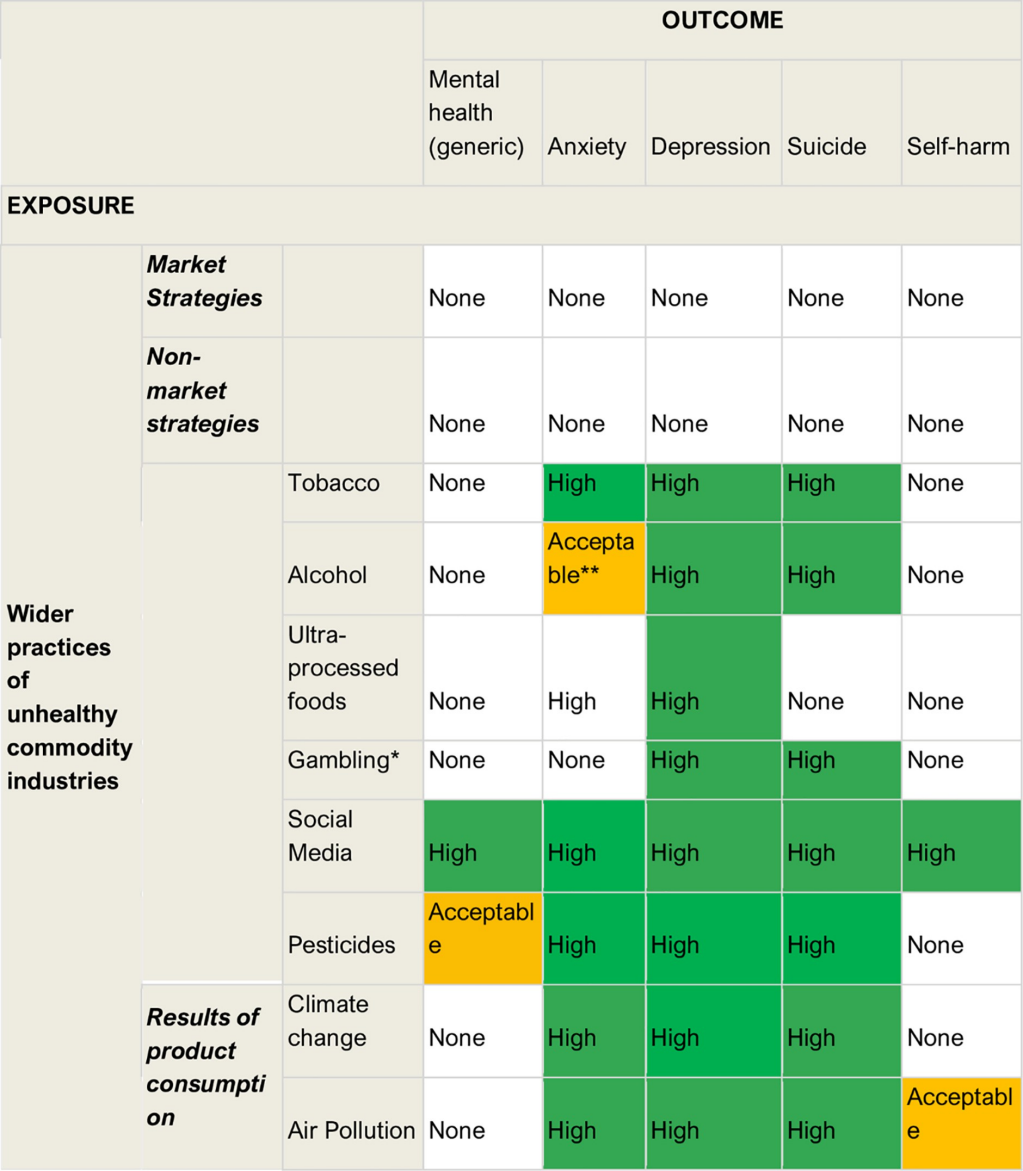
Highest quality of evidence for associations. This figure outlines the highest quality of review evidence found for each exposure and outcome (as assessed by the review authors). Green reflects high quality, amber acceptable quality and no colour reflects no studies found. *From individual studies (no pooled effect sizes) ** Outcome was offspring anxiety and depression following maternal prenatal exposure.

Fig 4 provides an overview of the highest quality of evidence (as rated by our reviewers) for positive relationships between respective exposures and outcomes. In summary, there is evidence from high quality reviews linking: tobacco, social media, UPF, pesticides, climate change and air pollution with anxiety; alcohol, tobacco, gambling, social media, ultra-processed foods, and air-pollution with depression; alcohol, tobacco, gambling, social media, climate change and air pollution with suicide; and social media with self-harm.

### Funding of reviews

Information was also collected on funding of reviews. Funding was not declared in 12 of the included reviews, 15 reported that they had received no funding, 4 that there were no competing financial interests (without outlining funders), and 2 that they had received funding from private foundations. None of the reviews declared any funding from industry but authors in 2 of the reviews reported they had previously received payment from pharmaceutical companies. The remaining 32 received funding from universities, governmental bodies, research organisations (e.g., Medical Research Council, NIHR) or international bodies (WHO and EU). None of the included reviews considered the funding of their included studies or stratified their results depending on industry funding.

## Discussion

To our knowledge, this is the first umbrella review examining the impact of commercial determinants on mental ill health. We found evidence from high quality reviews for associations between alcohol, tobacco, gambling, social media, ultra-processed foods, and air pollution with depression; alcohol, tobacco, gambling, social media, climate change and air pollution with suicide; climate change and air pollution with anxiety; and social media with self-harm. There was a lack of evidence examining wider practices of commercial industries and their impact on mental health.

We found evidence from high quality reviews for associations between five out of the six commodities examined and suicide [27, 40, 42, 45, 52, 62, 70, 76, 86] – the two highest quality reviews with alcohol as an exposure both found significant associations with completed suicide, for instance, while smoking reviews demonstrated a dose-response relationship with this outcome. One review [27] called for smoking to be included in risk assessments for suicide.

While suicide is clearly an extremely important outcome, it is important also to note the evidence base for other less serious but far more common mental health outcomes, such as anxiety and depression.

Overall, for exposures, there were fewer reviews identified for gambling and ultra-processed foods, with high heterogeneity of study design and definition of exposures. The studies included in the social media reviews were meanwhile mostly cross-sectional in design, with many using self-reported measures of both social media exposure and mental health outcomes, but there was high quality evidence for associations between social media use and depression, as well as suicide. The temporality of some of these relationships were, however, less clear than for other exposures (e.g., tobacco).

These identified gaps in the literature may, in part, reflect the well documented influence of industry on research, including research agendas [87, 88]. For example, the limited amount of review-level evidence on gambling and mental health, particularly of high-quality, is remarkable given the nature of these products and their impacts. In this respect, it is noteworthy that the gambling industry has been the main funder of such research in many countries for approximately 40 years [89, 90]. It was also striking that only one of the CDoH industries (alcohol) had been the focus of any research analysing relevant policies to tackle its impacts. It is also important to consider the wider mental health burden incurred by those close to someone affected by mental ill-health (for instance, the devastating impact of suicides on family and friend networks), a literature not included in this review. The mental health impacts on affected others should effectively be understood in the same way as other secondary exposures. Further consideration is similarly needed of the mental ill-health associated with the physical health impacts from these commercial determinants (e.g., lung cancer, liver cirrhosis, and violence). Finally, there are likely to also be mental ill-health burdens associated with wider harms such as the impact on communities from clustering of alcohol or gambling venues or the noise created by fossil-fuel based transport systems. Such considerations could inform future systems approaches to research on this topic and more comprehensive mappings of the commercial determinants of health.

### Suggested pathways

Several similarities were identified in the potential pathways by which consuming these products may impact on health. For example, for tobacco products, alcohol, and ultra-processed foods - most studies highlighted the role of inflammation. For products that are not directly consumed into the body, e.g., social media or gambling, the pathways for mental health outcomes may be via wider impacts such as relational or financial factors.

Yet this review also identified key gaps in the evidence base in relation to pathways around wider commercial impacts on mental health. Such an agenda will also require a shift in how harm - and pathways or chains of harm - are conceptualised, as well as greater appreciation of the differences between how health and disease impact on individuals versus populations. Further research should focus on wider systems, practices and pathways through which commercial actors influence physical and mental health.

### Methodological challenges

This review identified many methodological challenges in measuring commercial determinants of health. Even when considering relatively easier exposures, such as smoking or alcohol, there are substantial differences in approaches to measurement. It becomes more challenging still when considering more difficult exposures, such as air pollution and ultra-processed food consumption; when consumption is part of our everyday experience, it can be difficult to recall or measure accurately. For example, variable measurements were used for alcohol, including both self-reported unit intake and blood alcohol levels, while some studies used “ever use” of alcohol as an exposure. This latter measure obscures important insights given the substantial proportion of the population that has consumed alcohol at any point in their lives. Indeed, substantial methodological challenges are involved in measuring alcohol consumption, with intake often underreported and misremembered - alcohol consumed in the home, for instance, is unlikely to be served in recommended or standard measures. Social media was another exposure with specific methodological challenges. There are two components to social media that can be difficult to disentangle: (1) the *platforms* on which users can engage and the ways these platforms influence people’s use of social media and its roles in their lives, and (2) the *content* created by the users themselves to which others are then exposed.

Use of the term “problem” user was found across unhealthy commodity industries and was ill defined in all of these. Likewise, research has shown that terms such as “responsible drinking” are often poorly defined and can be used to provide pro industry framings of product harm [91]. The authors of one high-quality review [81] highlight key methodological challenges in the climate change/air pollution space, particularly due to the linking of events to climate change, the difficulties in temporal measurements before and after an event and the difficulty of controlling for confounders. Finally, it should also be noted that there is substantial overlap between different industries, even within our review; for example, the food industry is involved in pesticide usage as well as the production of highly processed food products.

### Strengths and limitations

This umbrella review is the first to consider the commercial determinants of mental ill health as a primary focus. In drawing together and mapping the evidence both for the unhealthy commodities and wider practices, we can identify where the evidence is strongest and where the evidence gaps are most clear. This makes this review useful for informing development of frameworks of mental health, as well as development of mental health policy.

Our study included both middle, and low-income countries in our search terms and so was not limited just to high income countries.

We made some changes to the review design following pilot searches. Firstly, we established it was not feasible to stratify the results by PROGRESS-Plus due to the number of studies identified, and substantial variations across the evidence base. Following reflection and discussion between the review group about the complexity of obesity-related research, including the confounding role of physical activity, it was agreed that obesity would no longer be included as an outcome. Addiction was also excluded as an outcome due to the large volume of search results returned that were judged not relevant for this review (e.g., focusing on the addictive properties of social media). Following discussion, in the context of this review, addiction seemed to be more of a confounder or a mediator of these relationships. In classing it as an outcome or an exposure, the focus of the review might therefore have shifted to examine whether products were addictive or if addiction itself was associated with mental ill health.

As we limited the included reviews to those that focused specifically on our exposures and outcomes of interest during the search, to avoid a large amount of irrelevant material, we may have excluded some additional findings that were not included in specific reviews. However, we felt that reviews specifically focused on our exposures and outcomes of interest were likely to include most of any relevant evidence. Finally, including only papers in the English language does mean that we could have missed findings from papers published in other languages. We were also unable to comprehensively examine inequalities in commercial determinants of mental ill health. This was due to both the number of papers available and the small number that considered differences in demographic groups. Although wider search terms were included for all industries, this review was also limited to six key industries. It did not include other key and interlinking industries such as the meat and dairy, chemical, beauty, and pharmaceutical industries. Many of these industries have overlapping practices and actors, so separating them can be challenging. For example, although online gambling was included, online gaming was mostly excluded, despite a large overlap between the two and the fact that many online gaming products include gambling elements. Examining the impact of fossil fuels was also particularly challenging – given, for instance, there are clear links with climate change and pollution, but fossil fuels are not the only cause. Finally, we restricted this umbrella review to include only reviews and not primary research papers. This may have led to the exclusion of relevant or new research (that is yet to be included in a review). Throughout the review, discussion with the wider review group aimed to guide these decisions. Overall, this umbrella review is the first that the authors are aware of that maps the evidence base for the ‘commercial determinants’ of mental health. While previous reviews have focused on individual unhealthy commodities (e.g., tobacco products), analysing these commodities within the context of a wider range of industries can lead to a greater understanding of the concept of CDoH, as well as the need to act across various industries and settings to reduce health-harming practices, and improve mental health.

## Conclusions

In conclusion, there is strong evidence that smoking, alcohol, and air pollution are associated with mental ill health. The evidence bases for ultra-processed foods, gambling, social media, and climate change are less developed but already include high-quality reviews demonstrating associations between these industries and various negative mental health outcomes. There is a striking lack of research examining the wider actions of corporations on mental health outcomes. Given these findings, commercial determinants should be routinely included within frameworks to examine and improve mental health.

## Supporting information

S1 Fig Search strategy Medline. Search strategy for our umbrella review using Medline.
(DOCX)

S2 Fig Search strategy Embase. Search strategy for our umbrella review using Embase.
(DOCX)

S3 Fig Search strategy PsychInfo. Search strategy for our umbrella review using PsychInfo.
(DOCX)
