# Supplementary material for: Commercial determinants of mental ill health: An umbrella review
Source: PLOS Glob Public Health. 2024 Aug 28;4(8):e0003605. doi: 10.1371/journal.pgph.0003605 (PMC11355563; doi:10.1371/journal.pgph.0003605)
Supplement: S1 Fig — Search strategy for our umbrella review using Medline. (DOCX) [file pgph.0003605.s001.docx]

**S1 Search strategy Medline.** Search strategy for our umbrella review using Medline.

|  | **SEARCH STRATEGY - MEDLINE** |
| --- | --- |
|  | exp Industry/ or Meat-Packing Industry/ or "Extraction and Processing Industry"/ or Manufacturing Industry/ or Textile Industry/ or Drug Industry/ or Tobacco Industry/ or "Oil and Gas Industry"/ or Food Industry/ or Construction Industry/ or Coal Industry/ or Chemical Industry/ or Book Industry/ or Food-Processing Industry/ |
| 2. | exp Commerce/ |
| 3. | exp Professional Corporations/ |
| 4. | exp Advertising/ |
| 5. | exp Marketing/ |
| 6. | exp Alcoholism/ or exp Alcohol Drinking/ or Binge Drinking/ or Alcoholic Intoxication/ |
| 7. | exp Tobacco Products/ |
| 8. | Tobacco/ or Smoking Prevention/ or Smoking Cessation/ or exp Smoking/ or Vaping/ or Nicotine/ |
| 9. | exp Gambling/ |
| 10. | exp Social Media/ |
| 11. | exp Fast Foods/ |
| 12. | exp Fossil Fuels/ |
| 13. | Obesity, Morbid/ or Obesity Management/ or exp Obesity/ or Pediatric Obesity/ or Obesity, Abdominal/ |
| 14. | Climate/ or exp Environmental Pollution/ or exp Climate Change/ or Greenhouse Effect/ |
| 15. | (((commerce or commercial or corporation* or corporate) adj3 health) or corporate social responsibility or industry or adverti#ing or advertisement or advert* or marketing or marketing strategies or alcohol* drinking or alcohol* beverages or binge drinking or wine or beer or sprit or liquor or tobacco or smok* or cigarette* or nicotine or e-cig* or vape* or vaping or gambling or gamble* or betting or social media or Facebook or twitter or Instagram or tiktok or ultra-processed food* or processed food* or junk food* or fossil fuel* or oil or non-renewable energy or natural gas or petroleum or coal or obesity or overweight or high BMI or climate change or global warming or global heating or greenhouse gas* or pollution or plastic or microplastic* or contamina*).mp. [mp=title, abstract, original title, name of substance word, subject heading word, floating sub-heading word, keyword heading word, organism supplementary concept word, protocol supplementary concept word, rare disease supplementary concept word, unique identifier, synonyms] |
| 16. | 1 or 2 or 3 or 4 or 5 or 6 or 7 or 8 or 9 or 10 or 11 or 12 or 13 or 14 or 15 |
| 17. | Mental Health/ |
| 18. | Mental Disorders/ |
| 19. | Mood Disorders/ |
| 20. | exp Depression/ |
| 21. | exp Anxiety/ |
| 22. | Suicide/ or Suicide, Completed/ or Suicide, Attempted/ |
| 23. | exp Self-Injurious Behavior/ |
| 24. | (((Anxiety or anxious or anxiety disorder or depress* or depressive disorder or mixed anxiety) and depressive disorder) or mood disorder or suicide or self-harm or mental ill health or mental health or mental health problems or mental illness or mental disorder or common mental disorder).mp. [mp=title, abstract, original title, name of substance word, subject heading word, floating sub-heading word, keyword heading word, organism supplementary concept word, protocol supplementary concept word, rare disease supplementary concept word, unique identifier, synonyms] |
| 25. | "Systematic Review"/ or "Review"/ or review.mp. |
| 26. | 17 or 18 or 19 or 20 or 22 or 23 or 24 |
| 27. | 16 and 26 |
| 28. | 25 and 27 |
| 29. | limit 28 to (english language and "review articles" and humans and yr="2012 -Current") |
