# Supplementary material for: Commercial determinants of mental ill health: An umbrella review
Source: PLOS Glob Public Health. 2024 Aug 28;4(8):e0003605. doi: 10.1371/journal.pgph.0003605 (PMC11355563; doi:10.1371/journal.pgph.0003605)
Supplement: S2 Fig — Search strategy for our umbrella review using Embase. (DOCX) [file pgph.0003605.s002.docx]

**S2 Search strategy Embase.** Search strategy for our umbrella review using Embase.

| 1. | meat industry/ or "industry and industrial phenomena"/ or plastic industry/ or food industry/ or wine industry/ or oil industry/ or petrochemical industry/ or exp industry/ or dairy industry/ or tobacco industry/ or offshore oil industry/ or chemical industry/ or sugar industry/ or automobile industry/ or leather industry/ |
| --- | --- |
| 2. | exp multinational corporation/ |
| 3. | exp commercial phenomena/ |
| 4. | exp advertizing/ or exp advertising/ |
| 5. | exp marketing/ or exp social marketing/ |
| 6. | exp alcohol/ or exp alcohol consumption/ or exp alcohol production/ or exp alcohol abuse/ |
| 7. | exp binge drinking/ or exp alcoholism/ or exp alcohol consumption/ or exp drinking behavior/ |
| 8. | exp smoking device/ or exp smoking/ or exp cigarette smoking/ or exp smoking habit/ or exp adolescent smoking/ |
| 9. | exp nicotine/ or exp vaping/ or exp electronic cigarette/ |
| 10. | exp pathological gambling/ or exp gambling/ |
| 11. | exp social media/ |
| 12. | exp processed food/ |
| 13. | exp fast food/ |
| 14. | exp greenhouse effect/ or exp climate change/ or exp fossil fuel/ |
| 15. | exp water pollution/ or exp radioactive water pollution/ or exp plastic pollution/ or exp "pollution and pollution related phenomena"/ or exp indoor air pollution/ or exp light pollution/ or exp radioactive air pollution/ or exp microplastic pollution/ or exp sea pollution/ or exp air pollution/ or exp pollution/ or exp soil pollution/ |
| 16. | childhood obesity/ or morbid obesity/ or abdominal obesity/ or diet-induced obesity/ or adolescent obesity/ or obesity/ |
| 17. | (((commerce or commercial or corporation* or corporate) adj3 health) or corporate social responsibility or industry or adverti#ing or advertisement or advert* or marketing or marketing strategies or alcohol* drinking or alcohol* beverages or binge drinking or wine or beer or sprit or liquor or tobacco or smok* or cigarette* or nicotine or e-cig* or vape* or vaping or gambling or gamble* or betting or social media or Facebook or twitter or Instagram or tiktok or ultra-processed food* or processed food* or junk food* or fossil fuel* or oil or non-renewable energy or natural gas or petroleum or coal or obesity or overweight or high BMI or climate change or global warming or global heating or greenhouse gas* or pollution or plastic or microplastic* or contamina*).mp. [mp=title, abstract, heading word, drug trade name, original title, device manufacturer, drug manufacturer, device trade name, keyword heading word, floating subheading word, candidate term word] |
| 18. | exp mental health/ |
| 19. | mental disease/ |
| 20. | mood disorder/ |
| 21. | exp anxiety disorder/ or Generalized Anxiety Disorder-7/ or exp anxiety/ or generalized anxiety disorder/ or "Hospital Anxiety and Depression Scale-Depression"/ or eco-anxiety/ or Generalized Anxiety Disorder Scale/ |
| 22. | major depression/ or "Hospital Anxiety and Depression Scale"/ or late life depression/ or exp depression/ or adolescent depression/ or "mixed anxiety and depression"/ or organic depression/ or Beck Depression Inventory/ or long term depression/ or Self-rating Depression Scale/ |
| 23. | exp automutilation/ |
| 24. | suicide attempt/ or exp suicide/ |
| 25. | (((Anxiety or anxious or anxiety disorder or depress* or depressive disorder or mixed anxiety) and depressive disorder) or mood disorder or suicide or self-harm or mental ill health or mental health or mental health problems or mental illness or mental disorder or common mental disorder).mp. [mp=title, abstract, heading word, drug trade name, original title, device manufacturer, drug manufacturer, device trade name, keyword heading word, floating subheading word, candidate term word] |
| 26. | exp review/ |
| 27. | exp meta analysis/ |
| 28. | exp "Systematic Review"/ |
| 29. | 1 or 2 or 3 or 4 or 5 or 6 or 7 or 8 or 9 or 10 or 11 or 12 or 13 or 14 or 15 or 16 or 17 |
| 30. | 18 or 19 or 20 or 21 or 22 or 23 or 24 or 25 |
| 31. | 26 or 27 or 28 |
| 32. | 29 and 30 and 31 |
| 33. | limit 32 to (human and english language and "systematic review" and yr="2012 -Current") |
