# Supplementary material for: Commercial determinants of mental ill health: An umbrella review
Source: PLOS Glob Public Health. 2024 Aug 28;4(8):e0003605. doi: 10.1371/journal.pgph.0003605 (PMC11355563; doi:10.1371/journal.pgph.0003605)
Supplement: S3 Fig — Search strategy for our umbrella review using PsychInfo. (DOCX) [file pgph.0003605.s003.docx]

**S3 Search strategy PsychInfo.** Search strategy for our umbrella review using PsychInfo.

| 1. | exp Business/ |
| --- | --- |
| 2. | exp Commerce/ |
| 3. | exp Television Advertising/ or exp Advertising/ |
| 4. | exp Marketing/ or exp Digital Marketing/ or exp Social Marketing/ |
| 5. | exp Alcohol Drinking Patterns/ or exp Alcoholism/ or exp Alcohol Abuse/ or exp Binge Drinking/ |
| 6. | exp Tobacco Smoking/ or exp Electronic Cigarettes/ or exp Nicotine/ |
| 7. | exp Gambling Disorder/ or exp Gambling/ |
| 8. | exp Social Media/ |
| 9. | exp Fast Food/ |
| 10. | exp Environmental Effects/ or exp Pollution/ or exp Climate Change/ or exp Business Organizations/ or exp Global Warming/ |
| 11. | (((commerce or commercial or corporation* or corporate) adj3 health) or corporate social responsibility or industry or adverti#ing or advertisement or advert* or marketing or marketing strategies or alcohol* drinking or alcohol* beverages or binge drinking or wine or beer or sprit or liquor or tobacco or smok* or cigarette* or nicotine or e-cig* or vape* or vaping or gambling or gamble* or betting or social media or Facebook or twitter or Instagram or tiktok or ultra-processed food* or processed food* or junk food* or fossil fuel* or oil or non-renewable energy or natural gas or petroleum or coal or obesity or overweight or high BMI or climate change or global warming or global heating or greenhouse gas* or pollution or plastic or microplastic* or contamina*).mp. [mp=title, abstract, heading word, table of contents, key concepts, original title, tests & measures, mesh word] |
| 12. | 1 or 2 or 3 or 4 or 5 or 6 or 7 or 8 or 9 or 10 or 11 |
| 13. | exp Mental Health/ |
| 14. | exp Mental Disorders/ |
| 15. | exp Affective Disorders/ |
| 16. | exp Anxiety Disorders/ or exp Anxiety/ or exp Generalized Anxiety Disorder/ |
| 17. | exp "Depression (Emotion)"/ or exp Major Depression/ or exp Reactive Depression/ or exp Recurrent Depression/ or exp Beck Depression Inventory/ |
| 18. | exp Self-Mutilation/ or exp Self-Destructive Behavior/ or exp Self-Inflicted Wounds/ or exp Self-Injurious Behavior/ or exp Attempted Suicide/ or exp Suicide/ or exp Suicidal Ideation/ |
| 19. | (((Anxiety or anxious or anxiety disorder or depress* or depressive disorder or mixed anxiety) and depressive disorder) or mood disorder or suicide or self-harm or mental ill health or mental health or mental health problems or mental illness or mental disorder or common mental disorder).mp. [mp=title, abstract, heading word, table of contents, key concepts, original title, tests & measures, mesh word] |
| 20. | exp "Systematic Review"/ or Systematic review.mp. or exp "Literature Review"/ |
| 21. | Meta-analysis.mp. or exp Meta Analysis/ |
| 22. | 20 or 21 |
| 23. | 13 or 14 or 15 or 16 or 17 or 18 or 19 |
| 24. | 12 and 22 and 23 |
| 25. | limit 24 to (human and english language and yr="2012 - 2022") |
